# Refractory Lupus Podocytopathy: The Role of Repeat Biopsy and Rituximab

**DOI:** 10.7759/cureus.91918

**Published:** 2025-09-09

**Authors:** Hui Yi Shan

**Affiliations:** 1 Medicine/Nephrology and Hypertension, Keck School of Medicine - University of Southern California, Los Angeles, USA

**Keywords:** lupus nephritis, lupus podocytopathy, podocytopathy, rituximab therapy, systemic lupus erythematosus

## Abstract

Lupus podocytopathy is a rare renal manifestation of systemic lupus erythematosus (SLE) that requires treatment strategies distinct from those used for proliferative lupus nephritis. In this report, the author describes a 28-year-old man with biopsy-proven class III lupus nephritis whose proteinuria progressed despite multiple induction regimens, including high-dose corticosteroids with mycophenolate mofetil, voclosporin, and cyclophosphamide-based therapy. A repeat kidney biopsy revealed the development of lupus podocytopathy with features of the focal segmental glomerulosclerosis variant. On the basis of these findings, he was treated with a rituximab biosimilar, which led to improvements in both serologic activity and proteinuria. This case highlights the diagnostic utility of repeat kidney biopsy in refractory disease and supports the use of rituximab or its biosimilars as an effective therapeutic option for steroid-resistant lupus podocytopathy.

## Introduction

Lupus podocytopathy accounts for approximately 1% of lupus nephritis biopsies [1]. This condition is not included in the International Society of Nephrology/Renal Pathology Society (ISN/RPS) classification system for lupus nephritis [2]. The main clinical manifestation of lupus podocytopathy is nephrotic syndrome, often in parallel with systemic disease activity and extrarenal involvement [1]. On kidney biopsy, the hallmark finding is diffuse podocyte foot process effacement, with absent or only mesangial immune complex deposition on electron microscopy [1,3]. This entity can be further subclassified into minimal change disease (MCD) and focal segmental glomerulosclerosis (FSGS) variants; the latter is associated with a higher risk of acute kidney injury, reduced responsiveness to therapy, and poorer outcomes [1,3]. As a distinct clinicopathologic entity, lupus podocytopathy differs in both presentation and prognosis from proliferative lupus nephritis and warrants a tailored therapeutic strategy. In this report, the author describes a case of refractory lupus nephritis that transformed into lupus podocytopathy, as identified on repeat kidney biopsy. The case underscores the importance of repeat biopsy in guiding management and discusses the use of rituximab, or its biosimilar, as an effective therapeutic option for steroid-resistant lupus podocytopathy.

## Case presentation

A 28-year-old man with a history of systemic lupus erythematosus (SLE) was hospitalized in October 2021 for lupus nephritis. At presentation, his serum creatinine was 0.7 mg/dL, and urinalysis showed 2+ protein, 2+ blood, 6-10 WBCs, and 3-10 RBCs. Proteinuria was 1.0 g/day. Renal biopsy revealed class III active lupus nephritis with mild-to-moderate foot process effacement on electron microscopy. He received pulse methylprednisolone 1 g IV daily for three days, followed by mycophenolate mofetil (MMF) 1,500 mg twice daily and a tapering course of prednisone. Although the anti-double-stranded DNA (dsDNA) antibody level declined significantly, there was no improvement in proteinuria. By March 2022, proteinuria had increased to 3.0 g/day, prompting escalation of prednisone to 60 mg daily and the addition of voclosporin 23.7 mg twice daily while maintaining MMF at 1,500 mg twice daily. His proteinuria improved to 1.4 g/day over three months but then worsened to 4.0 g/day by November 2022. His serum creatinine was 1.1 mg/dL. MMF was then replaced with cyclophosphamide (500 mg IV q2 weeks for six doses), without a meaningful reduction in proteinuria.

**Figure 1:**
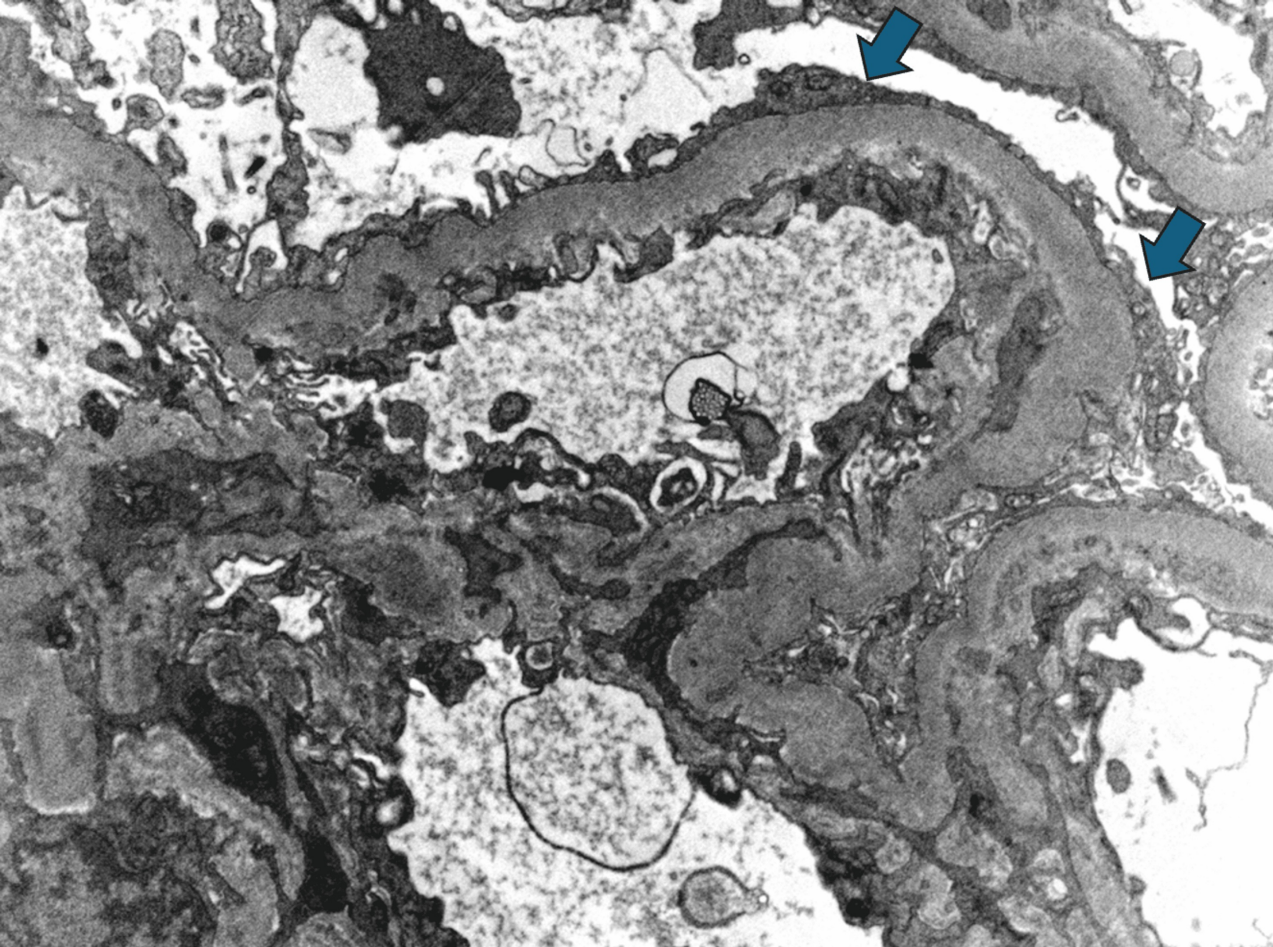
Patient’s renal biopsy finding showing diffuse podocyte foot process effacement (blue arrows) on electron microscopy.

**Figure 2:**
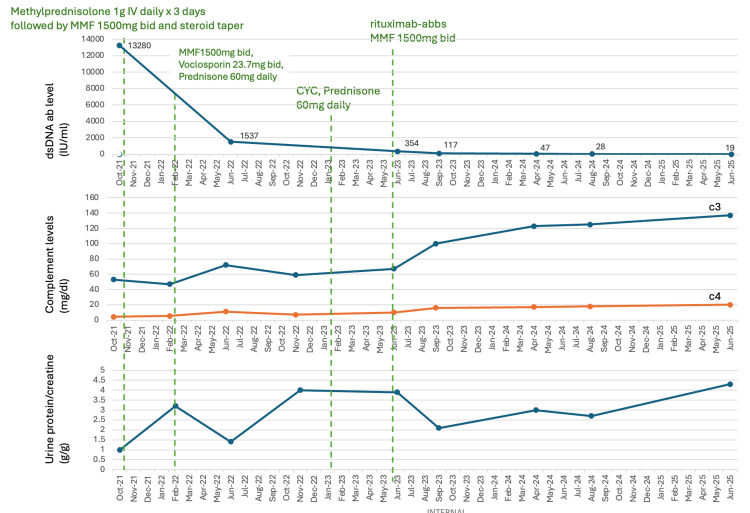
Serological and proteinuria trends in response to immunosuppressive regimens. MMF = Mycophenolate mofetil; CYC = Cyclophosphamide

In April 2023, a repeat renal biopsy demonstrated chronic diffuse glomerulonephritis with segmental sclerosis, consistent with class IV lupus nephritis with minimal activity. Electron microscopy revealed diffuse effacement of podocyte foot processes, and immune complex deposits were predominantly located in the mesangium, consistent with lupus podocytopathy (Figure 1). Given the patient’s persistent proteinuria despite high-dose prednisone and traditional induction therapies, rituximab-abbs (rituximab biosimilar) at 1 g IV two weeks apart for two doses was initiated in June 2023. This regimen produced further serological improvement in anti-dsDNA antibody level and achieved normalization of complement levels. Serum creatinine level stayed stable, ranging from 0.8 to 1.1 mg/dL. Proteinuria improved from 4.0 g to 2.0 g/day over five months. However, proteinuria began to rise again, while complement levels stayed normal and anti-dsDNA trended downward (Figure 2). A second course of rituximab is planned for this patient.

## Discussion

In 2016, Hu et al. reported the largest cohort of lupus podocytopathy to date, comprising 50 cases [3]. All biopsies demonstrated extensive foot process effacement, and mesangial electron-dense deposits were identified in 47 specimens. Every patient presented with nephrotic syndrome, with a median proteinuria of 5.72 g per 24 hours. Acute kidney injury was relatively uncommon overall (34%) but occurred more frequently in the FSGS subtype (78%) than in the MCD subtype. Complete remission was achieved in 92.3% of patients with MCD, compared with only 22% of those with FSGS. These findings were consistent with prior studies: patients with MCD subtype typically respond to a short course of high-dose glucocorticoids, whereas those with FSGS subtype require induction therapy combining glucocorticoids with additional immunosuppressive agents [3-5]. Relapse is common, affecting more than half of patients. Maintenance therapy with glucocorticoids alone resulted in relapse in 89.5% of cases, while combination maintenance therapy reduced the relapse rate by more than 50% [4].

The patient described in this case was initially diagnosed with class III lupus nephritis. His proteinuria was refractory to high-dose prednisone, MMF, cyclophosphamide, and adjunctive voclosporin. A repeat kidney biopsy helped identify the transformation from proliferative lupus nephritis to lupus podocytopathy with evidence of segmental sclerosis. The kidney biopsy results, together with his steroid resistance, support the diagnosis of the FSGS subtype of lupus podocytopathy.

Rituximab has been reported to show efficacy in treating refractory primary podocytopathies, including MCD and primary FSGS [6,7]. Recent advances in MCD pathogenesis have identified anti-nephrin antibodies as crucial pathogenic factors, and B-cell depletion with rituximab reduces autoantibody production. Beyond its immunological effects, rituximab exerts a direct protective effect on podocytes by binding to SMPDL-3b protein, thereby preventing cytoskeletal disruption [8]. Because of these reasons, a rituximab biosimilar was selected for this patient and yielded a good serological and proteinuria response.

The clinical efficacy of rituximab also depends on the duration and degree of B-cell depletion in specific patients. Substantial variability in peripheral B-cell depletion was observed in patients with lupus nephritis treated with rituximab [9]. In this patient, nephrotic-range proteinuria recurred after B-cell reconstitution, underscoring the need for repeated rituximab dosing to maintain remission.

## Conclusions

This case highlights several key principles in the management of SLE-related renal disease: (1) a repeat kidney biopsy in refractory disease is a valuable diagnostic tool that can guide therapeutic decisions; (2) lupus podocytopathy is a distinct entity requiring treatment strategies different from those for proliferative lupus nephritis; and (3) given its emerging role in the pathogenesis of primary podocytopathies, rituximab, or its biosimilar, represents a viable option for steroid-resistant lupus podocytopathy, with maintenance therapy often required to sustain remission.
